# Design, Synthesis and Antitumor Activity of Novel 4-Methyl-(3'*S*,4'*S*)-*cis*-khellactone Derivatives

**DOI:** 10.3390/molecules18044158

**Published:** 2013-04-08

**Authors:** Luhui Ren, Xue Du, Mengnan Hu, Chaoqun Yan, Taigang Liang, Qingshan Li

**Affiliations:** School of Pharmaceutical Science, Shanxi Medical University, No 56, Xinjian Nan Road, Taiyuan 030001, Shanxi, China; E-Mails: renluhui@126.com (L.R.); duxue_1988@163.com (X.D.); hmn0624@163.com (M.H.); chaoqunchaoqunyan@163.com (C.Y.)

**Keywords:** 4-methyl-(3'*S*,4'*S*)-*cis*-khellactone derivatives, asymmetric synthesis, antitumor activity

## Abstract

An asymmetric synthesis of a series of novel 4-methyl-(3'*S*,4'*S*)-*cis*-khellactone derivatives **3a**–**o** is reported for the first time. Their structures were confirmed by ^1^H-NMR, ^13^C-NMR and MS. Their cytotoxic activity was evaluated by the MTT assay against three selected human cancer cell lines: HEPG-2 (human liver carcinoma), SGC-7901 (human gastric carcinoma), LS174T (human colon carcinoma). Some compounds showed high inhibitory activity against these human cancer cell lines. Among them, compound **3a** exhibited strong cytotoxicity, with IC_50_ values ranging from 8.51 to 29.65 μM. The results showed that 4-methyl-*cis*-khellactone derivatives with *S*,*S* configuration could be a potential antitumor agents.

## 1. Introduction

**Figure 1:**
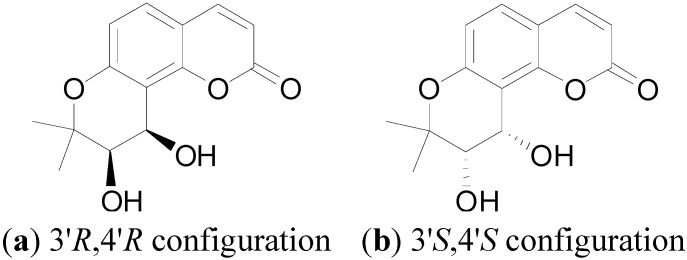
Structures of *cis*-khellactone with (**a**) 3'*R*,4'*R* or (**b**) 3'*S*,4'*S* configuration.

Khellactone coumarins, which constitute a small branch of the coumarin family, are notable because of their extensive bioactivities, including anti-HIV [1], anti-platelet aggregation [2], calcium antagonist activity [3], P-glycoprotein inhibitory ability, *etc*. [4,5]. Particullarly, the famous compound DCK [3'R,4'R-di-O-(camphanoyl-(+)-cis-khellactone] in this class, along with its derivatives, have received increasing attention due to their potent anti-HIV activity [6]. As shown in Figure 1, khellactone coumarins contain two chiral carbons, C-3' and C-4', and most of the reported compounds possess 3'*R*,4'*R* configuration. Lots of literature has shown that the rigid stereochemistry of 3'*R* and 4'*R*-configured khellactone derivatives is crucial for anti-HIV activity [7,8,9]. 

Khellactone coumarins with 3'*S*,4'*S* configuration exist mainly in the plants *Peucedanum praeruptorum* Dunn and *Peucedanum japonicum* [10]. Recently, more and more researchers have paisd close attention to the calcium antagonist activity and P-glycoprotein inhibitory ability of the (3'*S*,4'*S*)-*cis*-khellactone coumarins [11,12], whereas, other activities of (3'*S*,4'*S*)-*cis*-khellactone coumarins, for example, antitumor activity, have been rarely reported. 

**Figure 2:**
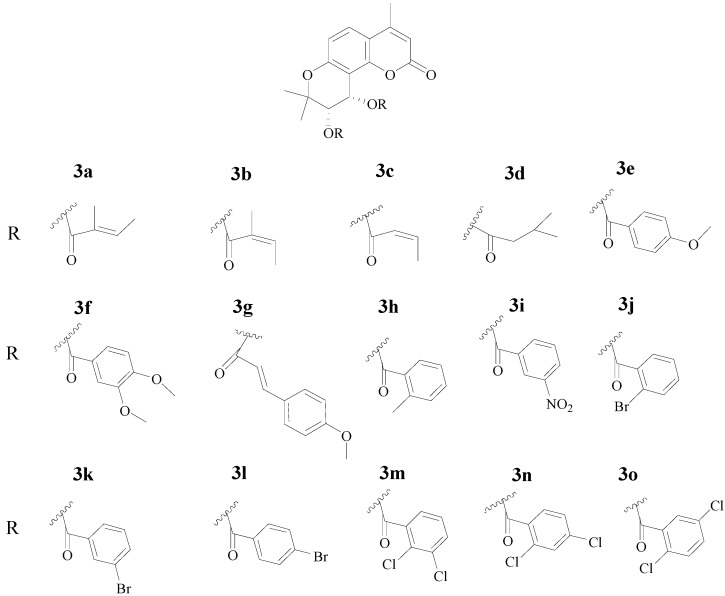
Structures of 4-methyl-(3'*S*,4'*S*)-*cis*-khellactone derivatives **3a**–**o**.

In the present study, a series of 4-methyl-(3'*S*,4'*S*)-*cis*-khellactone derivatives **3a**–**o** (Figure 2) were designed and asymmetrically synthesized. Furthermore, all the synthesized compounds were screened against three cultured human cancer cell lines (HEPG-2, SGC-7901, LS174T). The results showed that some compounds showed potent cytotoxicity and compound **3a** in particular exhibited the most significant cytotoxicity against these cancer cell lines, especially against HEPG-2 cells. 

## 2. Results and Discussion

### 2.1. Chemistry

**Scheme 1:**
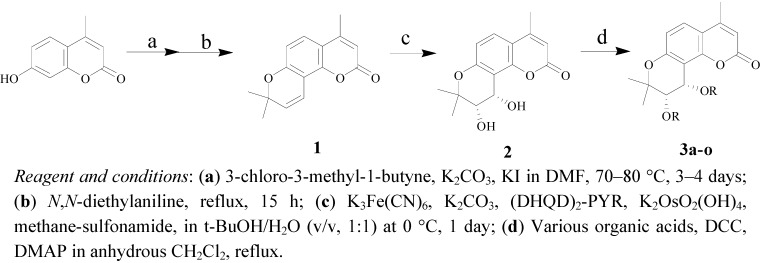
Synthesis of (3'*S*,4'*S*)-4-methyl-*cis*-khellactone derivatives **3a**–**o**.

Our synthetic strategy was first to obtain 4-methylseselin (**1**), and then to stereoselectively synthesize 4-methyl-(−)-*cis*-khellactone (**2**) and its derivatives **3a**–**o**. The corresponding synthetic routes are shown in Scheme 1. 

*Reagent and conditions*: (**a**) 3-chloro-3-methyl-1-butyne, K_2_CO_3_, KI in DMF, 70–80 °C, 3–4 days; (**b**) *N*,*N*-diethylaniline, reflux, 15 h; (**c**) K_3_Fe(CN)_6_, K_2_CO_3_, (DHQD)_2_-PYR, K_2_OsO_2_(OH)_4_, methane-sulfonamide, in t-BuOH/H_2_O (v/v, 1:1) at 0 °C, 1 day; (**d**) Various organic acids, DCC, DMAP in anhydrous CH_2_Cl_2_, reflux.

7-Hydroxy-4-methylcoumarin, a commercially available compound, was reacted with 3-chloro-3-methyl-1-butyne in DMF in the presence of anhydrous potassium carbonate and potassium iodide and then thermal rearrangement occurred in boiling diethylaniline to form 4-methylseselin (**1**) by following the procedures published previously [13,14]. 

4-Methylseselin **(1)** was asymmetrically dihdroxylated using (DHQD)_2_-PYR (hydroquinidine 2,5-diphenyl-4,6-pyrimidinediyl diether) as a chiral catalyst to give 4-methyl-(−)-*cis*-khellactone (**2**) [15]. Without further purification, compound **2** was directly esterified in anhydrous CH_2_Cl_2_ with various organic acids in the presence of dimethylaminopyridine (DMAP) and *N,N'*-dicyclohexylcarbodiimide (DCC), respectively, to produce 4-methyl-*cis*-khellactone derivatives **3a**–**o ** [16]. 

**Scheme 2:**
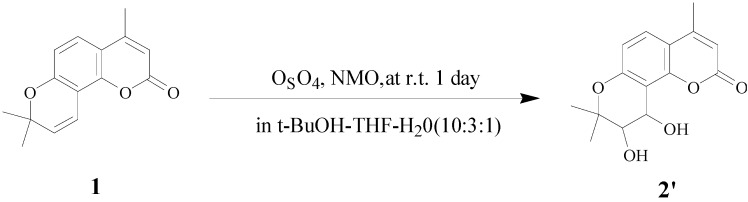
Syntheses of 4-methyl-(±)-*cis*-khellactone **2'**.

For the determination of enantiomeric excess, 4-methylseselin **(1)** was oxidized with OsO_4_ to give racemic 4-methyl-*cis*-khellactones **2'** [17] (Scheme 2). The asymmetric dihydroxylation for **2** is highly stereoselective, with good enantiomeric excess (74% e.e.) by chiral HPLC analysis. (DHQD)_2_-PYR leads primarily to the *cis*-diol with *S, S* configuration [15]. 

### 2.2. *In Vitro* Biological Evaluation

All of the 4-methyl-(3'*S*,4'*S*)-cis-khellactone derivatives were evaluated for cytotoxic activity *in vitro* against three human cancer cell lines (HEPG-2, SGC-7901, LS174T) using the MTT assay. The antitumor activity was indicated in terms of IC_50_ (μM) values and the results are presented in Table 1.

**Table 1:** Cytotoxic activity of the synthesized compounds against three human cancer cell lines ^a^.

**Compound**	**IC_50_ ± SD (μM)**		
	**HEPG-2 **	**SGC-7901**	**LS174T**
**3a**	8.51 ± 3.03	29.65 ± 6.12	19.14 ± 3.68
**3b**	23.64 ± 5.29	59.81 ± 9.85	47.17 ± 6.17
**3c**	15.62 ± 4.15	29.36 ± 6.55	20.16 ± 4.79
**3d**	78.23 ± 11.07	>100	>100
**3e**	50.92 ± 8.01	78.93 ± 16.27	54.79 ± 9.45
**3f**	67.54 ± 8.36	>100	55.20 ± 12.10
**3g**	89.29 ± 17.81	71.30 ± 12.76	>100
**3h**	22.69 ± 5.90	31.14 ± 4.32	19.60 ± 4.32
**3i**	>100	75.23 ± 8.09	>100
**3j**	>100	89.57 ± 11.92	91.13 ± 13.05
**3k**	>100	>100	84.03 ± 9.98
**3l**	60.92 ± 8.73	22.64 ± 5.44	38.51 ± 4.63
**3m**	77.55 ± 10.60	82.09 ± 13.76	>100
**3n**	86.97 ± 11.09	>100	>100
**3o**	>100	90.36 ± 15.27	>100

^a^ The data represented the mean of three experiments in triplicate and were expressed as means ± SD.

As shown in Table 1, some compounds showed promising anticancer activity for certain cancer cell lines *in vitro* and the changes of 3' and 4' substituents on the pyran ring had a significant influence on the cytotoxicity. For example, both compounds **3a** and **3c** exhibited potent inhibitory effects on the indicated cell lines, meanwhile, **3a** with tigloyl group at 3' and 4' position showed the most significant cytotoxicity against the HEPG-2 cell lines, with an IC_50_ value of 8.51 μM. Compound **3b**, compared to **3a**, displayed low cytotoxicity against these cell lines, especially against the SGC-7901 and LS174T cell lines. The results revealed that the *Z* isomer of the 2-methyl-2-butenoyl group is superior to the *E* isomer for the antitumor activity of these two compounds. Additionally, compound **3h** with an *o*–methylbenzoyl group showed noteworthy inhibitory activity against all three human cancer cell lines among all the synthesized compounds with aromatic groups at the 3' and 4' positions. For SGC-7901 cells, the most highly active compound was **3l** with a *p*-bromobenzoyl group, which had an IC_50_ value of 22.64 μM. The preliminary results revealed that the tigloyl group at the 3' and 4' position on the pyran ring was favorable structural moiety to retain the anticancer activity. Further investigation to search for more potent groups is under way.

## 3. Experimental

### 3.1. General

All chemicals were purchased from Aladdin Chemicals Co. (Shanghai, China) and Energy Chemicals Co. (Shanghai, China), whereas chiral catalyst (DHQD)_2_-PYR was obtained from Sigma-Aldrich (St. Louis, MO, USA). The reactions were monitored by thin-layer chromatography (TLC) with silica gel GF254 plates (Qingdao Jiyida Silica Reagent Manufacture, Qingdao, China), which were visualized by UV light. Melting points were measured on a XT-4 melting point apparatus (Shanghai Precision Scientific Instrument Co. Ltd., Shanghai, China) without correction. The ^1^H-NMR spectra were acquired on Bruker Avance 600 MHz spectrometer (Bruker Corporation, Karlsruhe, Germany) from solutions in either deuterated chloroform or deuterated dimethylsulfoxide (DMSO-d_6_) containing tetramethylsilane as internal reference, while the ^13^C-NMR spectra were recorded at 150 MHz. ESI mass spectra were obtained on an API QTRAP 3200 LC-MS spectrometer (AB SCIEX Corporation, Boston, MA, USA). Optical rotations were measured on a Perkin–Elmer 241 polarimeter (PE Corporation, Nolwalk, CT, USA) at room temperature. The enatiomeric excesses (*ee* values) of the compounds were determined by Chiralpak AS-H chiral HPLC analysis by using a Shimadzu LC-10A instrument (Shimadzu, Suzhou, China), using *n*-hexane/2-propanol as eluent.

### 3.2. Procedure for the Synthesis of 4-Methylseselin (**1**)

To a solution of 4-methyl-7-hydroxycoumarin (1.76 g, 10 mmol), K_2_CO_3_ (3.45 g, 25 mmol), KI (1.66 g, 10 mmol) in DMF (20 mL) was added excess 3-chloro-3-methyl-1-butyne (6 mL), then the mixture was heated to 70–80 °C for 3–4 days. After the solid K_2_CO_3_ was filtered, the brown filtrate was poured into EtOAc and washed with water three times and dried over anhydrous Na_2_SO_4_. The solvent was removed *in vacuo*. The residue, without purification, was directly heated to reflux in 20 mL of *N,N*-diethylaniline for 15 h. The reaction mixture was cooled to room temperature, poured into EtOAc, and washed with 10% aqueous HCl, water and brine. The organic layer was separated, and solvent was removed *in vacuo*. The residue was purified by column chromatography with an eluant of petroleum ether-EtOAc = 10:1 to afford compound **1**. Molecular formula (MW): C_15_H_14_O_3_ (242.27 g/mol); white solid; 23% Yield; mp: 139–141 °C (lit. [7]. 141–143 °C); ^1^H-NMR (DMSO-d_6_) δ 1.43 (6H, s, 2 × C-2'-CH_3_), 2.38 (3H, s, C-4-CH_3_), 5.93 (1H, d, *J* = 10.04 Hz, H-3'), 6.23 (1H, s, H-3), 6.74 (1H, d, *J* = 10.04 Hz, H-4'), 6.82 (1H, d, *J* = 8 .70 Hz, H-6), 7.54 (1H, d, *J* = 8.70 Hz, H-5); MS (ESI) *m/z* 264.9 ([M+Na]^+^).

### 3.3. Procedure for the Synthesis of 4-Methyl-(-)-cis-khellactone (**2**)

K_3_Fe(CN)_6_ (246 mg, 0.75 mmol) and K_2_CO_3_ (105 mg, 0.75 mmol) were dissolved in *t*-BuOH/H_2_O (1:1 v/v, 5 mL) at r.t. Then, hydroquinidine 2,5-diphenyl-4,6-pyrimidinediyl diether [(DHQD)_2_ -PYR], 4.4 mg, 0.005 mmol) and K_2_OsO_2_(OH)_4_ (2 mg, 0.005 mmol) were added to the solution. The mixture was stirred for 15 min. Then, the solution was cooled to 0 °C and methane-sulfonamide (24 mg, 0.25 mmol) added under stirring. When the solution turned from a light yellow to an orange color, compound **1** (61 mg, 0.25 mmol) was added. The mixture was stirred at 0 °C for 1 day. The reaction was monitored using TLC, and at completion, Na_2_S_2_O_5_ (1 g), water (2.5 mL), and CH_2_Cl_2_ (2.5 mL) were added. After being stirred for 4 h at room temperature, the mixture was extracted with CH_2_Cl_2_ three times. The combined organic layer was dried over Na_2_SO_4_, and then solvent was removed to afford crude compound **2**. The crude compound was crystallized from petroleum ether/acetone to give pure **2**. Molecular formula (MW): C_15_H_16_O_5_ (276.28 g/mol); white solid; 70% Yield; mp: 213–215 °C; [α]D^20^ = –59.5 (*c* 0.1, DMSO); 74% ee. HPLC: Chiralpak AS-H (hexane/i-PrOH, 90:10, flow rate 0.8 mL/min, wavelength = 323 nm), t_R_ (minor) = 26.71 min, t_R_ (major) = 68.88 min; ^1^H-NMR (DMSO-d_6_) δ 1.35 (3H, s, C-2'-CH_3_), 1.37 (3H, s, C-2'-CH_3_), 2.37 (3H, s, C-4-CH_3_), 3.61 (1H, br, H-4'), 4.90 (1H, br, H-3'), 5.14 and 5.21 (each 1H, br, OH × 2), 6.19 (1H, s, H-3), 6.77 (1H, d, *J* = 8 .75 Hz, H-6), 7.57 (1H, d, *J* = 8.75 Hz, H-5); ^13^C-NMR (DMSO-d_6_) δ 18.17, 20.98, 26.86, 60.23, 71.23, 78.68, 110.55, 111.71, 112.56, 113.42, 125.66, 153.28, 153.52, 155.58, 160.03; MS (ESI) *m/z* 277.09 ([M+H]^+^).

### 3.4. Procedure for the Synthesis of 4-Methyl-(3'S,4'S)-cis-Khellactone Derivatives **3a**–**o**

To a solution of crude compound **2** (138 mg, 0.5 mmol) in anhydrous CH_2_Cl_2_ (10 mL) tiglic acid (6 mmol), angelic acid (6 mmol), crotonic acid (6 mmol), isovaleric acid (4 mmol), 4-methoxybenzoic acid (3 mmol), 3,4-dimethoxybenzoic acid (3 mmol), 4-methoxycinnamic acid (2 mmol), *o*-toluic acid (3 mmol), 3-nitrobenzoic acid (3 mmol), 2-bromobenzoic acid (3 mmol), 3-bromobenzoic acid (3 mmol), 4-bromobenzoic acid (3 mmol), 2,3-dichlorobenzoic acid (2 mmol), 2,4-dichlorobenzoic acid (2 mmol) or 2,5-dichlorobenzoic acid (2 mmol) were added, respectively, followed by *N,N’*-dicyclo-hexylcarbodiimide (412 mg, 2 mmol) and 4-dimethylaminopyridine (8 mg, 0.064 mmol). The mixture was heated to reflux until the reaction was complete as monitored by TLC. After cooling to room temperature, filtered, and the filtrate was separated and purified by column chromatography (petroleum ether/acetone, 10:1) to give pure target compounds **3a**–**o**.

*(3'S,4'S)-Di-O-tigloyl-4-methyl-(+)-cis-khellactone* (**3a**). Molecular formula (MW): C_25_H_28_O_7_ (440.49 g/mol); white solid; 23% Yield; mp: 84–86 °C; [α${]}_{D}^{20}$: +3.8 (c 0.1, CH_2_Cl_2_). ^1^H-NMR (DMSO-d_6_) δ 1.39 (3H, s, C-2'-CH_3_), 1.43 (3H, s, C-2'-CH_3_), 1.71 (3H, br s, H-5''), 1.72 (3H, br s, H-5'''), 1.74 (3H, br d, H-4''), 1.76 (3H, br d, H-4'''), 2.39 (3H, s, C-4-CH_3_), 5.32 (1H, d, *J* = 4.92 Hz, H-3'), 6.21 (1H, s, H-3), 6.50 (1H, d, *J* = 4.92 Hz, H-4'), 6.66 (1H, m, H-3''), 6.72 (1H, m, H-3'''), 6.94 (1H, d, *J* = 8.86 Hz, 7.72 (1H, d, *J* = 8.86 Hz, H-5), H-6); ^13^C-NMR (CDCl_3_) δ 11.87, 12.05, 14.25, 14.33, 18.63, 22.27, 25.40, 60.64, 70.50, 77.36, 107.48, 112.06, 113.51, 113.92, 125.91, 127.96, 128.31, 137.08, 138.16, 152.04, 153.46, 156.47, 159.72, 166.50, 166.56; MS (ESI) *m/z* 462.7,463.9 ([M+Na]^+^).

*(3'S,4'S)-Di-O-angeloyl-4-methyl-(+)-cis-khellactone* (**3b**). Molecular formula (MW): C_25_H_28_O_7_ (440.49 g/mol); white solid; 31% Yield; mp: 151–153 °C; [α${]}_{D}^{20}$: +4.2 (c 0.1, CH_2_Cl_2_). ^1^H-NMR (CDCl_3_) δ 1.42 (3H, s, C-2'-CH_3_), 1.48 (3H, s, C-2'-CH_3_), 1.73 (3H, br d, H-4''), 1.75 (3H, br d, H-4'''), 1.78 (3H, br s, H-5''), 1.80 (3H, br s, H-5'''), 2.36 (3H, s, C-4-CH_3_), 5.39 (1H, d, *J* = 4.92 Hz, H-3'), 6.08 (1H, s, H-3), 6.65 (1H, d, *J* = 4.92 Hz, H-4'), 6.74 (1H, m, H-3''), 6.78 (1H, m, H-3'''), 6.83 (1H, d, *J* = 8.84 Hz, H-6), 7.49 (1H, d, *J* = 8.84 Hz, H-5); ^13^C-NMR (CDCl_3_) δ 11.91, 12.08, 14.28, 14.35, 18.65, 22.40, 25.41, 60.76, 70.54, 77.47, 107.60, 112.16, 113.58, 113.93, 125.87, 128.04, 128.41, 137.09, 138.16, 151.96, 153.58, 156.57, 159.74, 166.55, 166.62; MS (ESI) *m/z* 462.9 ([M+Na]^+^). 

*(3'S,4'S)-Di-O-crotonoyl-4-methyl-(+)-cis-khellactone* (**3c**). Molecular formula (MW): C_23_H_24_O_7_ (412.43 g/mol); white solid; 45% Yield; mp: 63–65 °C; [α${]}_{D}^{20}$: +5.1 (c 0.1, CH_2_Cl_2_). ^1^H-NMR (CDCl_3_) δ 1.40 (3H, s, C-2'-CH_3_), 1.45 (3H, s, C-2'-CH_3_), 1.83 (3H, m, H-4'''), 1.85 (3H, m, H-4''), 2.35 (3H, s, C-4-CH_3_), 5.36 (1H, d, *J* = 4.88 Hz, H-3'), 5.78–5.84 (2H, m, H-2'', H-2'''), 6.07 (1H, s, H-3), 6.60 (1H, d, *J* = 4.88 Hz, H-4'), 6.82 (1H, d, *J* = 8.84 Hz, H-6), 6.90–6.98 (2H, m, H-3'', H-3'''), 7.49 (1H, d, *J* = 8.84 Hz, H-5); ^13^C-NMR (CDCl_3_) δ 17.98, 18.00, 18.64, 22.27, 25.23, 60.52, 70.20, 77.41, 107.23, 112.05, 113.50, 113.94, 121.79, 122.03, 125.95, 145.18, 145.96, 152.09, 153.41, 156.46, 159.73, 164.91, 165.06; MS (ESI) *m/z* 434.9,435.8 ([M+Na]^+^).

*(3'S,4'S)-Di-O-isovaleryloxy-4-methyl-(+)-cis-khellactone* (**3d**). Molecular formula (MW): C_25_H_32_O_7_ (444.52 g/mol); light yellow oil; 52% Yield; [α${]}_{D}^{20}$: +4.5 (c 0.1, CH_2_Cl_2_). ^1^H-NMR (CDCl_3_) δ 0.95–0.98 (12H, m, CH_3_×4), 1.41 (3H, s, C-2'-CH_3_), 1.43 (3H, s, C-2'-CH_3_), 2.10–2.30 (6H, m, COCH_2_ × 2, CH × 2), 2.37 (3H, s, C-4-CH_3_), 5.31 (1H, d, *J* = 4.79 Hz, H-3'), 6.10 (1H, s, H-3), 6.54 (1H, d, *J* = 4.79 Hz, H-4'), 6.81 (1H, d, *J* = 8.85 Hz, H-6), 7.49 (1H, d, *J* = 8.85 Hz, H-5); ^13^C-NMR (CDCl_3_) δ 18.67, 22.36, 22.41, 25.31, 25.43, 25.47, 43.05, 43.23, 60.49, 70.37, 107.24, 112.03, 113.50, 113.94, 125.98, 152.13, 153.32, 156.36, 159.72, 171.78; MS (ESI) *m/z* 467.3 ([M+Na]^+^).

*(3'S,4'S)-Di-O-p-methoxybenzoyl-4-methyl-(-)-cis-khellactone* (**3e**). Molecular formula (MW): C_31_H_28_O_9_ (544.55 g/mol); white solid; 19% Yield; mp: 177–179 °C; [α${]}_{D}^{20}$: −35.9 (c 0.1, CH_2_Cl_2_). ^1^H-NMR (CDCl_3_) δ 1.49 (3H, s, C-2'-CH_3_), 1.62 (3H, s, C-2'-CH_3_), 2.37 (3H, s, C-4-CH_3_), 3.82 (3H, s, OCH_3_), 3.83 (3H, s, OCH_3_), 5.64 (1H, d, *J* = 4.92 Hz, H-3'), 6.06 (1H, s, H-3), 6.79–6.82(4H, m, Ar-H), 6.90 (1H, d, *J* = 8.88 Hz, H-6), 6.92 (1H, d, *J* = 4.92 Hz, H-4'), 7.54 (1H, d, *J* = 8.88 Hz, H-5), 7.81–7.86 (4H, m, Ar-H); ^13^C-NMR (CDCl_3_) δ 18.67, 22.44, 25.62, 55.35, 55.40, 61.01, 70.93, 77.53, 107.49, 112.25, 113.50, 113.62, 113.99, 121.83, 122.40, 126.09, 131.84, 131.89, 151.88, 153.63, 156.60, 159.58, 163.29, 163.60, 164.88, 164.96; MS (ESI) *m/z* 566.8 ([M+Na]^+^).

*(3'S,4'S)-bis-O-(3,4-Dimethoxybenzoyl)-4-methyl-(-)-cis-khellactone* (**3f**). Molecular formula (MW): C_33_H_32_O_11_ (604.60 g/mol); white solid; 28% Yield; mp : 207–209 °C; [α${]}_{D}^{20}$: −10.0 (c 0.1, CH_2_Cl_2_). ^1^H-NMR (CDCl_3_) δ 1.51 (3H, s, C-2'-CH_3_), 1.65 (3H, s, C-2'-CH_3_), 2.38 (3H, s, C-4-CH_3_), 3.66 (3H, s, OCH_3_), 3.76 (3H, s, OCH_3_), 3.89 (6H, s, OCH_3_), 5.65 (1H, d, *J* = 4.92 Hz, H-3'), 6.08 (1H, s, H-3), 6.76 (1H, d, *J* = 8.49 Hz, Ar-H), 6.79(1H, d, *J* = 8.49 Hz, Ar-H), 6.91 (1H, d, *J* = 8.90 Hz, H-6), 6.95 (1H, d, *J* = 4.92 Hz, H-4'), 7.38 (1H, d, *J* = 1.92 Hz, Ar-H), 7.43 (1H, d, *J* = 1.92 Hz, Ar-H), 7.50 (1H, m, Ar-H), 7.55 (1H, d, *J* = 8.90 Hz, H-5), 7.56 (1H, m, Ar-H); ^13^C-NMR (CDCl_3_) δ 18.66, 22.25, 25.73, 55.59, 55.82, 55.96, 60.98, 71.02, 77.40, 107.33, 110.22, 112.19, 112.23, 112.45, 113.60, 114.00, 121.79, 122.41, 123.71, 123.92, 126.19, 129.70, 129.96, 148.62, 151.97, 153.00, 153.31, 153.58, 156.55, 159.63, 164.85, 164.89; MS (ESI) *m/z* 626.8 ([M+Na]^+^).

*(3'S,4'S)-bis-O-p-Methoxycinnamoyl-4-methyl-(-)-cis-khellactone* (**3g**). Molecular formula (MW): C_35_H_32_O_9_ (596.62 g/mol); white solid; 34% Yield; mp: 120–122 °C; [α${]}_{D}^{20}$: −59.5 (c 0.1, CH_2_Cl_2_). ^1^H-NMR (CDCl_3_) δ 1.47 (3H, s, C-2'-CH_3_), 1.56 (3H, s, C-2'-CH_3_), 2.37 (3H, s, C-4-CH_3_), 3.81 (3H, s, OCH_3_), 3.82 (3H, s, OCH_3_), 5.51 (1H, d, *J* = 5.00 Hz, H-3'), 6.10 (1H, s, H-3), 6.31 (2H, d, *J* = 15.93 Hz, 2 × ArCH=), 6.77 (1H, d, *J* = 5.00 Hz, H-4'), 6.82-6.85 (4H, m, Ar-H), 6.88 (1H, d, *J* = 8.92 Hz, H-6), 7.37–7.41 (4H, m, Ar-H), 7.52 (1H, d, *J* = 8.92 Hz, H-5), 7.62 (2H, d, *J* = 15.93 Hz, 2 × -COCH=); ^13^C-NMR (CDCl_3_) δ 18.70, 22.67, 25.22, 55.31, 55.34, 60.82, 70.35, 77.63, 107.39, 112.20, 113.29, 113.43, 113.65, 113.98, 114.16, 114.25, 114.53, 114.99, 125.96, 126.98, 127.26, 129.82, 129.96, 132.33, 132.41, 145.07, 145.70, 151.97, 153.56, 156.59, 159.75, 161.29, 161.53, 165.72, 166.02; MS (ESI) *m/z* 618.8 ([M+Na]^+^).

*(3'S,4'S)-Di-O-o-methylbenzoyl-4-methyl-(-)-cis-khellactone* (**3h**). Molecular formula (MW): C_31_H_28_O_7_ (512.55 g/mol); white solid; 47% Yield; mp: 196–198 °C; [α${]}_{D}^{20}$: −36.7 (c 0.1, CH_2_Cl_2_). ^1^H-NMR (CDCl_3_) δ 1.54 (3H, s, C-2'-CH_3_), 1.61 (3H, s, C-2'-CH_3_), 2.38 (3H, s, C-4-CH_3_), 2.43 (3H, s, CH_3_), 2.52 (3H, s, CH_3_), 5.66 (1H, d, *J* = 4.95 Hz, H-3'), 6.09 (1H, s, H-3), 6.89 (1H, d, *J* = 8.88 Hz, H-6), 6.95 (1H, d, *J* = 4.95 Hz, H-4'), 7.06 (1H, m, Ar-H), 7.13 (2H, m, Ar-H), 7.20 (1H, m, Ar-H), 7.30 (1H, m, Ar-H), 7.36 (1H, m, Ar-H), 7.53 (1H, d, *J* = 8.88 Hz, H-5), 7.65 (1H, m, Ar-H), 7.80 (1H, m, Ar-H); ^13^C-NMR (CDCl_3_) δ 18.69, 20.92, 21.56, 22.48, 25.61, 60.99, 71.01, 77.43, 107.41, 112.18, 113.64, 114.04, 125.31, 125.58, 126.11, 128.74, 129.61, 129.87, 130.50, 131.36, 131.57, 131.71, 132.24, 140.53, 140.94, 152.04, 153.55, 156.54, 159.66, 165.96, 166.25; MS (ESI) *m/z* 534.5 ([M+Na]^+^).

*(3'S,4'S)-Di-O-m-nitrobenzoyl-4-methyl-(-)-cis-khellactone* (**3i**). Molecular formula (MW): C_29_H_22_N_2_O_11_ (574.49 g/mol); white solid; 34% Yield; mp: 187–189 °C; [α${]}_{D}^{20}$: −10.0 (c 0.1, CH_2_Cl_2_). ^1^H-NMR (CDCl_3_) δ 1.54 (3H, s, C-2'-CH_3_), 1.65 (3H, s, C-2'-CH_3_), 2.40 (3H, s, C-4-CH_3_), 5.74 (1H, d, *J* = 4.95 Hz, H-3'), 6.07 (1H, s, H-3), 6.96 (1H, d, *J* = 8.93 Hz, H-6), 6.98 (1H, d, *J* = 4.95 Hz, H-4'), 7.58–7.63 (2H, m, Ar-H), 7.61 (1H, d, *J* = 8.93 Hz, H-5), 8.27 (2H, m, Ar-H), 8.39 (2H, m, Ar-H), 8.56 (1H, m, Ar-H), 8.60 (1H, m, Ar-H); ^13^C-NMR (CDCl_3_) δ 18.78, 22.78, 25.04, 62.48, 71.82, 77.30, 105.94, 112.22, 113.93, 114.21, 124.30, 124.46, 126.76, 127.69, 128.00, 129.78, 129.91, 130.71, 131.07, 135.41, 135.71, 148.07, 148.21, 152.29, 153.42, 156.40, 159.47, 163.46, 163.57; MS (ESI) *m/z* 596.7 ([M+Na]^+^).

*(3'S,4'S)-Di-O-o-bromobenzoyl-4-methyl-(-)-cis-khellactone* **(3j)**. Molecular formula (MW): C_29_H_22_Br_2_O_7_ (642.29 g/mol); white solid; 42% Yield; mp: 178–180 °C; [α${]}_{D}^{20}$: −62.1 (c 0.1, CH_2_Cl_2_). ^1^H-NMR (600 MHz, CDCl_3_) δ 1.59 (3H, s, C-2'-CH_3_), 1.60 (3H, s, C-2'-CH_3_), 2.39 (3H, s, C-4-CH_3_), 5.73 (1H, d, *J* = 4.51 Hz, H-3'), 6.12 (1H, s, H-3), 6.87 (1H, d, *J* = 8.57 Hz, H-6), 6.94 (1H, d, *J* = 4.51 Hz, H-4'), 7.22 (1H, d, *J* = 7.31 Hz, Ar-H), 7.25 (1H, d, *J* = 7.31 Hz, Ar-H), 7.31 (2H, m, Ar-H), 7.53 (2H, m, Ar-H), 7.61 (1H, d, *J* = 7.57 Hz, Ar-H), 7.71 (1H, d, *J* = 7.57 Hz, Ar-H), 7.84 (1H, d, *J* = 8.57 Hz, H-5); ^13^C-NMR (150 MHz, CDCl_3_) δ 18.73, 22.25, 25.97, 62.13, 72.11, 77.45, 106.84, 112.19, 113.61, 114.12, 120.96, 121.41, 126.42, 127.06, 127.20, 131.03, 131.77, 132.05, 132.10, 132.68, 132.76, 133.74, 134.08, 152.16, 153.48, 156.43, 159.61, 165.20, 165.67; MS (ESI) *m/z* 662.0, 664.0, 666.5 ([M+Na]^+^).

*(3'S,4'S)-Di-O-m-bromobenzoyl-4-methyl-(-)-cis-khellactone* **(3k)**. Molecular formula (MW): C_29_H_22_Br_2_O_7_ (642.29 g/mol); white solid; 36% Yield; mp: 201–203 °C; [α${]}_{D}^{20}$: −17.8 (c 0.1, CH_2_Cl_2_). ^1^H-NMR (CDCl_3_) δ 1.51 (3H, s, C-2'-CH_3_), 1.63 (3H, s, C-2'-CH_3_), 2.39 (3H, s, C-4-CH_3_), 5.66 (1H, d, *J* = 5.00 Hz, H-3'), 6.09 (1H, s, H-3), 6.92 (1H, d, *J* = 8.52 Hz, H-6), 6.93 (1H, d, *J* = 5.00 Hz, H-4'), 7.24–7.27 (2H, m, Ar-H), 7.58 (1H, d, *J* = 8.52 Hz, H-5), 7.63–7.68 (2H, m, Ar-H), 7.81–7.86 (2H, m, Ar-H), 7.95 (2H, m, Ar-H); ^13^C-NMR (CDCl_3_) δ 18.70, 22.49, 25.36, 61.70, 71.46, 77.31, 106.59, 112.31, 113.80, 114.08, 122.38, 122.53, 126.47, 128.35, 128.46, 129.96, 130.02, 131.08, 131.54, 132.55, 132.64, 135.97, 136.37, 152.01, 153.52, 156.45, 159.46, 164.04, 164.07; MS (ESI) *m/z* 662.1, 664.3, 666.5 ([M+Na]^+^).

*(3'S,4'S)-Di-O-p-bromobenzoyl-4-methyl-(-)-cis-khellactone* (**3l**). Molecular formula (MW): C_29_H_22_Br_2_O_7_ (642.29 g/mol); white solid; 40% Yield; mp: 192–194 °C; [α${]}_{D}^{20}$: −20.0 (c 0.1, CH_2_Cl_2_). ^1^H-NMR (CDCl_3_) δ 1.50 (3H, s, C-2'-CH_3_), 1.60 (3H, s, C-2'-CH_3_), 2.38 (3H, s, C-4-CH_3_), 5.64 (1H, d, *J* = 4.82 Hz, H-3'), 6.08 (1H, s, H-3), 6.91 (1H, d, *J* = 8.98 Hz, H-6), 6.92 (1H, d, *J* = 4.82 Hz, H-4'), 7.49 (4H, m, Ar-H), 7.56 (1H, d, *J* = 8.98 Hz, H-5), 7.72 (4H, m, Ar-H); ^13^C-NMR (CDCl_3_) δ 18.70, 22.49, 25.36, 61.68, 71.35, 77.35, 106.71, 112.24, 113.76, 114.13, 126.41, 128.10, 128.21, 128.52, 128.62, 129.62, 131.17, 131.57, 131.68, 131.81, 132.15, 152.17, 153.48, 156.49, 159.67, 164.60, 164.69; MS (ESI) *m/z* 662.6, 664.5, 666.5 ([M+Na]^+^).

*(3'S,4'S)-bis-O-(2,3-dichlorobenzoyl)-4-methyl-(-)-cis-khellactone* (**3m**). Molecular formula (MW): C_29_H_20_Cl_4_O_7_ (622.28 g/mol); white solid; 29% Yield; mp: 197–199 °C; [α${]}_{D}^{20}$: −34.6 (c 0.1, CH_2_Cl_2_). ^1^H-NMR (CDCl_3_) δ 1.55 (3H, s, C-2'-CH_3_), 1.61 (3H, s, C-2'-CH_3_), 2.40 (3H, s, C-4-CH_3_), 5.71 (1H, d, *J* = 4.88 Hz, H-3'), 6.14 (1H, s, H-3), 6.88 (1H, d, *J* = 8.85 Hz, H-6), 6.92 (1H, d, *J* = 4.88 Hz, H-4'), 7.18 (1H, m, Ar-H), 7.26 (1H, m, Ar-H), 7.49 (1H, m, Ar-H), 7.55 (1H, d, *J* = 8.85 Hz, H-5), 7.58 (1H, m, Ar-H), 7.61 (1H, m, Ar-H), 7.72 (1H, m, Ar-H); ^13^C-NMR (CDCl_3_) δ 18.73, 21.90, 25.99, 62.44, 72.49, 77.28, 106.58, 112.19, 113.67, 114.23, 126.60, 127.32, 127.36, 128.63, 129.67, 130.62, 131.19, 132.41, 132.60, 133.29, 133.45, 133.95, 134.33, 152.33, 153.42, 156.38, 159.66, 164.55, 165.12; MS (ESI) *m/z* 642.1, 644.2 ([M+Na]^+^).

*(3'S,4'S)-bis-O-(2,4-dichlorobenzoyl)-4-methyl-(-)-cis-khellactone* (**3n**). Molecular formula (MW): C_29_H_20_Cl_4_O_7_ (622.28 g/mol); white solid; 41% Yield; mp: 140–142 °C; [α${]}_{D}^{20}$: −73.1 (c 0.1, CH_2_Cl_2_). ^1^H-NMR (CDCl_3_) δ 1.56 (3H, s, C-2'-CH_3_), 1.57 (3H, s, C-2'-CH_3_), 2.39 (3H, s, C-4-CH_3_), 5.69 (1H, d, *J* = 5.18 Hz, H-3'), 6.12 (1H, s, H-3), 6.88 (1H, d, *J* = 5.18 Hz, H-4'), 6.89 (1H, d, *J* = 8.95 Hz, H-6), 7.21 (1H, m, Ar-H), 7.27 (1H, m, Ar-H), 7.37 (1H, *J* = 1.92 Hz, Ar-H), 7.44 (1H, *J* = 1.92 Hz, Ar-H), 7.55 (1H, d, *J* = 8.95 Hz, H-5), 7.71 (1H, d, *J* = 8.45 Hz, Ar-H), 7.82 (1H, d, *J* = 8.45 Hz, Ar-H); ^13^C-NMR (CDCl_3_) δ 18.72, 22.23, 25.65, 62.28, 72.12, 77.28, 106.57, 112.19, 113.67, 114.14, 126.50, 127.02, 127.12, 127.69, 128.69, 130.51, 130.89, 132.24, 133.11, 134.21, 134.73, 138.00, 138.71, 152.22, 153.44, 156.38, 159.53, 163.86, 164.38; MS (ESI) *m/z* 642.0, 644.2 ([M+Na]^+^).

*(3'S,4'S)-bis-O-(2,5-dichlorobenzoyl)-4-methyl-(-)-cis-khellactone* (**3o**). Molecular formula (MW): C_29_H_20_Cl_4_O_7_ (622.28 g/mol); white solid; 28% Yield; mp: 116–118 °C; [α${]}_{D}^{20}$: −71.2 (c 0.1, CH_2_Cl_2_). ^1^H-NMR (CDCl_3_) δ 1.58 (6H, s, 2 × C-2'-CH_3_), 2.40 (3H, s, C-4-CH_3_), 5.69 (1H, d, *J* = 4.95 Hz, H-3'), 6.14 (1H, s, H-3), 6.90 (1H, d, *J* = 8.88 Hz, H-6), 6.91 (1H, d, *J* = 4.95 Hz, H-4'), 7.29–7.40 (4H, m, Ar-H), 7.57 (1H, d, *J* = 8.88 Hz, H-5), 7.72 (1H, d, *J* = 2.30 Hz, Ar-H), 7.81 (1H, d, *J* = 2.30 Hz, Ar-H); ^13^C-NMR (CDCl_3_) δ 18.73, 22.17, 25.70, 62.32, 72.32, 106.38, 112.28, 113.73, 114.12, 126.61, 130.61, 131.08, 131.27, 131.72, 131.75, 131.87, 131.92, 132.13, 132.34, 132.60, 132.82, 152.13, 153.45, 156.33, 159.44, 163.56, 163.87; MS (ESI) *m/z* 642.0, 644.2 ([M+Na]^+^).

### 3.5. Procedure for the Synthesis of 4-methyl-(±)-cis-Khellactone (2')

Compound **1** (0.242 g, 1 mmol) was added to a solution of osmium tetroxide (10 mg, 0.04 mmol) and *N*-methylmorpholine-*N*-oxide monohydrate (0.129 g, 1.1 mmol) in *t*-BuOH-THF-H_2_O (10:3:1, 10 mL) and the mixture was stirred for 1 day at r.t. Saturated NaHSO_3_ solution (80 mL) was added, the mixture kept stirring for 2 h and extracted with CH_2_C1_2_ (2 × 40 mL) and purified by column chromatography (petroleum ether/acetone, 5:1) to afford the pure compound **2'**. Molecular formula (MW) : C_15_H_16_O_5_ (276.28 g/mol); white solid; 56% Yield; MS (ESI) *m/z* 277.09 ([M+H]^+^).

### 3.6. *In Vitro* Biological Evaluation

All the cell lines were cultured in Dulbecco’s modified Eagle’s medium (DMEM), supplemented with 10% (v/v) heat-inactivated fetal bovine serum (FBS), 100 units/mL penicillin, and 100 µg/mL streptomycin in a humidified incubator aerated with 5% CO_2_ at 37 °C. The MTT assay was used to evaluate the cytotoxic activities *in vitro*. Briefly, cells were harvested during logarithmic growth phase and seeded in 96 well plates at a density of 2 × 10^4^ cells per well. After 24 h incubation at 37 °C in 5% CO_2_ atmosphere, cells were exposed to title compounds at concentrations from 5 to 200 μM for 48 h. Subsequently, MTT (25 μL, 5 mg/mL) was added to each well and the plates were incubated for a further 4 h at 37 °C. Then, the media were removed from all the wells and the resultant MTT formazan was solubilized in DMSO (200 μL). The absorbance values at 490 nm were measured using a microplate reader (Model 680, Bio-Rad, Hercules, CA, USA). The IC_50_ was calculated from the semi-logarithmic dose-response curves.

## 4. Conclusions

In conclusion, a series of novel 4-methyl-(3'*S*,4'*S*)-*cis*-khellactone derivatives were designed and asymmetrically synthesized. Antitumor evaluation indicated that some of these new compounds displayed high inhibitory activity against three human cancer cell lines. Among them, compound **3a** with a tigloyl group at the 3',4' positions on the pyran ring exhibited the most potent cytotoxic activity and these results could be useful for the further modification to obtain more potent antitumor agents.
